# Relationship between subscapularis tears and injuries to the biceps pulley

**DOI:** 10.1007/s00167-016-4374-9

**Published:** 2016-11-15

**Authors:** Arnaud Godenèche, Laurent Nové-Josserand, Stéphane Audebert, Bruno Toussaint, Patrick J. Denard, French Society for Arthroscopy (SFA), Alexandre Lädermann

**Affiliations:** 1 Centre Orthopédique Santy, Hôpital Privé Jean Mermoz, Ramsay Générale de Santé, 24 Avenue Paul Santy, 69008 Lyon, France; 2 Clinique du Cambresis, Cambrai, France; 3 Clinique Générale, Annecy, France; 4 Southern Oregon Orthopedics, Medford, OR USA; 5 Department of Orthopaedics and Rehabilitation, Oregon Health & Science University, Portland, OR USA; 6 Division of Orthopaedics and Trauma Surgery, La Tour Hospital, Rue J.-D. Maillard 3, 1217 Meyrin, Switzerland; 7 Faculty of Medicine, University of Geneva, Rue Michel-Servet 1, 1211 Geneva 4, Switzerland; 8 Division of Orthopaedics and Trauma Surgery, Department of Surgery, Geneva University Hospitals, Rue Gabrielle-Perret-Gentil 4, 1211 Geneva 14, Switzerland

**Keywords:** Shoulder surgery, Pulley, Subscapularis tear, Long-head biceps dislocation, Rotator cuff lesion, Diagnosis, Arthroscopy

## Abstract

**Purpose:**

The purpose of this study was to analyse the relationship between long head of the biceps brachii (LHBT) lesions and subscapularis tears. The hypothesis was that a bicipital pulley might remain intact, even in the case of a subscapularis tear.

**Methods:**

Between 2010 and 2011, all patients who had a primary arthroscopic repair of a subscapularis tear were potentially included in this prospective study. The outcome of interest was the prevalence and type of arthroscopic lesions of the LHBT and bicipital pulley. Furthermore, the supposed pathomechanics of injury and the treatment proposed (conservative, pulley repair, tenodesis, tenotomy, etc.) was recorded. The following baseline characteristics were assessed: age, sex, shoulder side, and limb dominance.

**Results:**

Of the 218 patients, the superior glenohumeral ligament/coracohumeral ligament (SGHL/CHL) complex was normal in 54 patients (25%), stretched in 84 patients (39%), and absent in 77 patients (35%). Below the SGHL/CHL complex in the bicipital groove, the medial wall of the LHBT sheath was normal in 25%, partially torn in 39%, and completely torn in 35%. In 25 of the 218 patients (11%), a pathologic LHBT with an intact SGHL/CHL complex was observed. In these cases, the medial wall of the bicipital sheath was torn in 92%.

**Conclusions:**

The biceps pulley system, including the SGHL/CHL complex and subscapularis tendon, merits recognition as an important anatomical structure, and its lesions contribute to shoulder pathology. The subscapularis tendon is very important for the stability of the LHBT and should be included in the pulley system. In cases of a tear associated with a lesion of the SGHL/CHL complex, the LHBT is nearly always unstable and pathologic.

**Level of evidence:**

II.

## Introduction

Effective diagnostic shoulder arthroscopy requires a thorough inspection of all anatomical areas implicated in shoulder pain and dysfunction [31]. The long head of the biceps brachii (LHBT) tendon is one such structure frequently implicated in shoulder pathology. The LHBT originates at the superior glenoid and travels intra-articularly before entering into the bicipital groove. These two anatomical locations are frequent sites of pathology and are important to examine. While the shape of the groove contributes to stability of the LHBT, the most important stabilizers are the superior glenohumeral ligament (SGHL), the coracohumeral ligament (CHL), and the interwoven fibres of the subscapularis and supraspinatus [16, 30]. The complex SGHL/CHL and the subscapularis tendon are intimately associated at their insertions onto the lesser tuberosity. This close association is likely the reason why these structures can simultaneously tear away from the humerus and yet remain attached to one another. An understanding of the associations of lesions in this area is crucial as sole evaluation of the subscapularis may be insufficient to decide whether a procedure on the long head of the biceps is required. However, the prevalence of simultaneous lesions of the aforementioned structures is not yet clearly defined in the literature [2].

The purpose of this study was to analyse the relationship between long head of the LHBT lesions and subscapularis tears.

## Materials and methods

Between 2010 and 2011, all patients with a subscapularis tear diagnosed by one surgeon of the French Arthroscopic Society were considered potentially eligible for inclusion in this prospective study. Inclusion criteria were (1) patients with subscapularis lesions that could be either isolated or associated with a partial [9] or small (<1 cm) [3] supraspinatus tear and (2) a complete and analysable recorded video of the surgery. Partial thickness supraspinatus tears were measured with a graduated probe and graded according to Ellman; Grade 1 measured less than 3 mm deep, Grade 2 were between 3 and 6 mm deep, depth not exceeding one-half of the tendon thickness, and, in Grade 3, the tear excessed 6 mm deep. We excluded patients with subscapularis lesions associated with a complete supraspinatus lesion (large or massive according to Cofield) [3] because they are associated with another type of “comma sign” (from Dilisio and Neyton) [8] in which the comma represents a subscapularis horizontal cleavage tear that assumes a vertical orientation because of the resultant superomedial vector on the tissue from the retracted supraspinatus tear.

A standard arthroscopy was performed and recorded with a 30° or a 70° arthroscope. Four senior surgeons (AG, NJL, TB, and SA) analysed the video data of all procedures. A training session to determine the key points that have to be passed in review was completed before evaluation of the images and videos. Discrepancies regarding video analysis were rectified by mutual review and agreement.

The type of subscapularis tear was analysed according to Toussaint et al. [28]. In this classification, four patterns were identified: type 1 is defined as an normal medial sling wall with partial subscapularis tendon detachment, type 2 is characterized by a combined partial subscapularis separation from the lesser tuberosity and a partial tear in the medial sling wall, type 3 is a complete subscapularis tendon detachment and a complete tear in the medial sling wall, with the most superficial fibres remaining continuous with the sling, and type 4 corresponds to a complete detachment of the subscapularis tendon from the humerus with a full-thickness tear, leaving a free lateral edge. The subscapularis retraction in the frontal plane was classified according to Patte: [22] in stage 1 the proximal stump was close to bony insertion, in stage 2 the stump was at level of humeral head, and stage 3 corresponded to retraction to the glenoid.

The type of SGHL and CHL lesions were assessed as normal, stretched/elongated, or torn. The prevalence of a “comma sign” of Lo and Burkhart [17], which corresponds to an arc formed by a portion of the SGHL/CHL complex and from the supraspinatus, which inserts on the superior–lateral aspect of the subscapularis tendon rather than to bone [29] was determined. The LHBT was assessed dynamically for stability (centred, subluxated if reached the summit of the medial wall of the bicipital groove, dislocated) and macroscopic aspect (normal, tendinosis, torn). Below the SGHL/CHL complex, in the bicipital groove, we analysed the soft tissue medial wall of the bicipital sheath (normal, partially torn, completely torn) either with a 70° scope [24] or after opening the sheath from the subacromial space [15].

The outcome of interest was the prevalence and type of arthroscopic lesions of the LHBT and bicipital pulley. Furthermore, the supposed pathomechanics of injury and the treatment proposed (conservative, pulley repair, tenodesis, tenotomy, etc.) was recorded. The following baseline characteristics were assessed: age, sex, shoulder side, and limb dominance.

The study protocol was approved by the institutional review board of the ethical committee of the Hôpital Privé Jean Mermoz, the Centre Orthopédique Santy approved this study (study 2015-12), and all patients gave informed written consent.

### Statistical analysis

The descriptive analysis consisted of frequencies and percentages for discrete data. Means and standard deviations were used for continuous data. Because this study was an anatomical observation study without a comparison group, we did not perform statistical comparisons or a priori power analysis.

## Results

During the study period, 406 consecutive patients underwent arthroscopic treatment of a subscapularis tear. A total of 186 patients were excluded from the study because of an associated complete rupture of the supraspinatus. Two patients declined to participate. Thus, there were 218 patients (218 shoulders) for the analysis. The demographic data and the type of subscapularis and supraspinatus lesions are summarized in Table 1.

**Table 1:** Demographic data

	*N* = 218
Male/female (%)	123 (56 %)/95 (44 %)
Mean age ± SD (range)	53 ± 9 years (30–75)
Side involved right/left	196/22
Dominant side (%)	61%
Type of subscapularis tear in %	
1	6
2	47.2
3	22.9
4	23.4
Not documented	0.5
Subscapularis retraction in %	
Patte 1	76.6
Patte 2	17
Patte 3	6.4
Comma sign (%)	
Yes	34.4
No	65.1
Not documented	0.5
Type of supraspinatus tear in %	
Intact	24.3
Ellman I	14.2
Ellman II	13.3
Ellman III	5
Small full thickness	41.7
Not documented	1.4

*SD* standard deviation

**Fig. 1:**
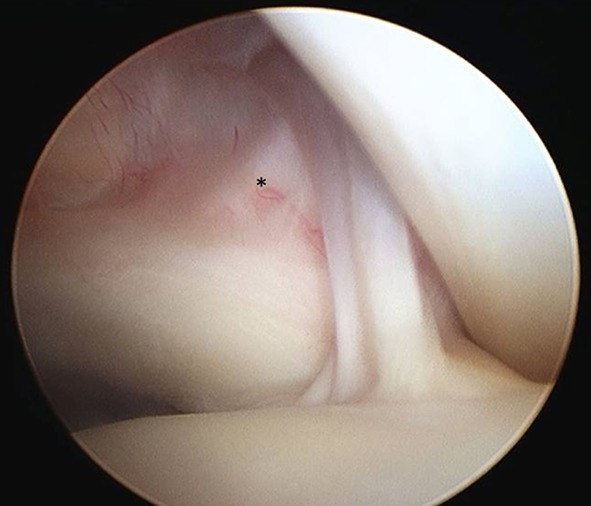
Right subscapularis tendon and biceps pulley in normal arthroscopic anatomy. Fibres of the SGHL/CHL complex (*asterisk*) inserted onto the subscapularis tendon

**Fig. 2:**
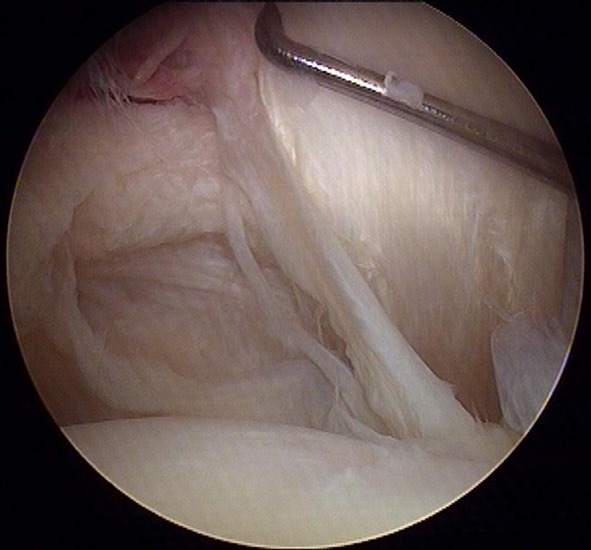
Biceps pulley present but stretched in case of torn subscapularis tendon associated with pathologic LHBT

**Fig. 3:**
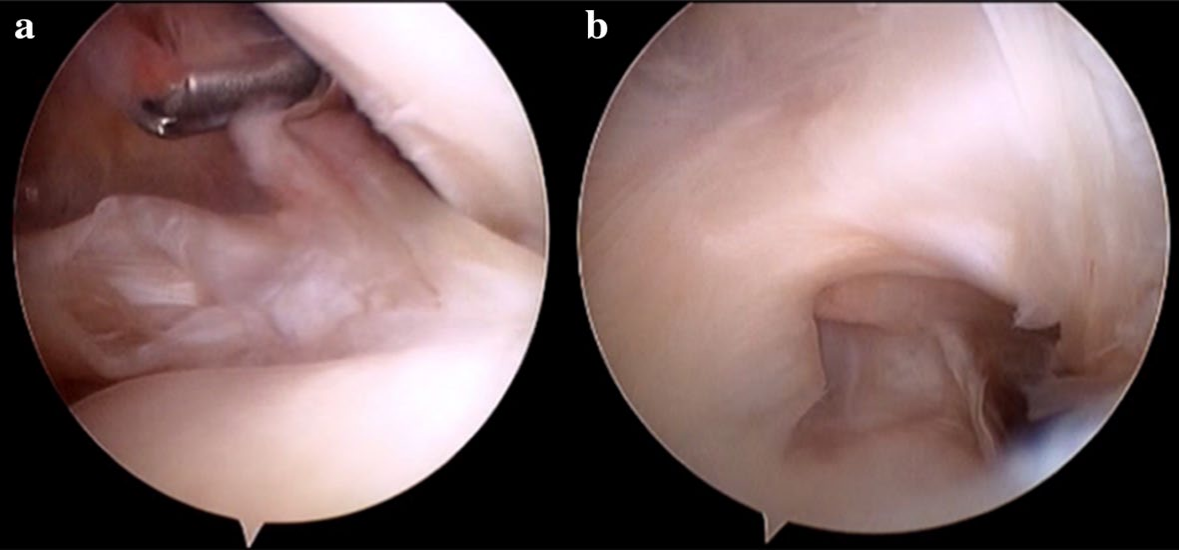
Right shoulder with posterior viewing portal. **a** Intact SGHL/CHL complex associated with partial deep subscapularis tear; **b** intra-articular view of a lesion of the medial wall of the intra-tubercular groove with a tear communicating with the subscapularis footprint

**Fig. 4:**
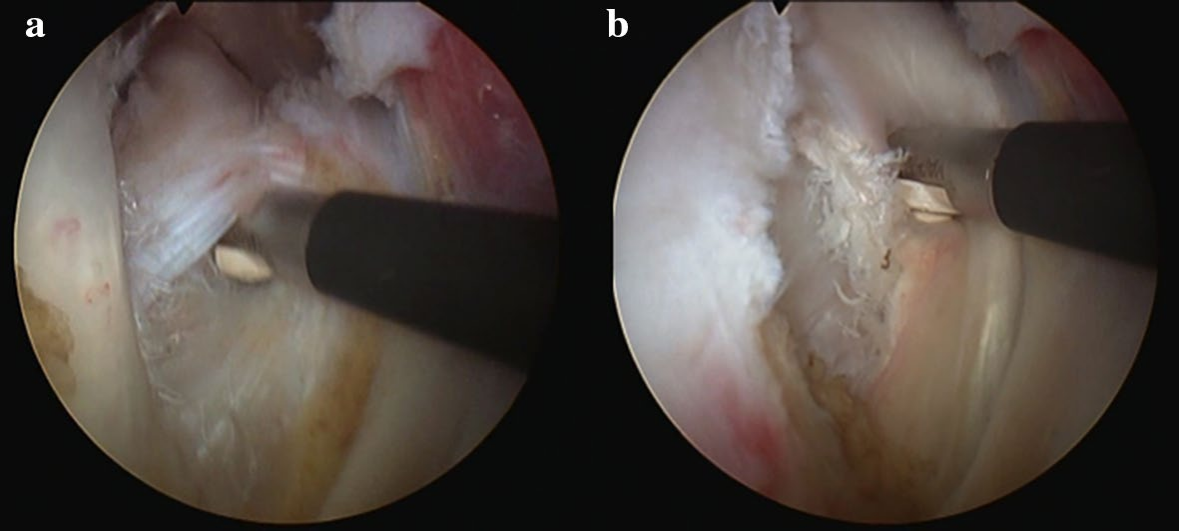
Extra-articular view of a right lesion of the medial wall of the intertubercular groove **a** with partial subscapularis tear, instability, and severe tendinosis of LHBT **b**. The SGHL/CHL complex and the aspect of intra-articular subscapularis tendon were normal

Evaluations of the biceps, of the bicipital sheath, and of the reflection pulley are summarized in Table 2. Of the 218 patients, the SGHL/CHL complex was normal in 54 patients (25%) (Fig. 1), stretched in 84 patients (39%) (Fig. 2), and torn in 77 patients (35%) (not documented in 3 cases). The data regarding the relationships between the SGHL/CHL and the LHBT lesions are summarized in Tables 3 and 4. Below the SGHL/CHL complex in the bicipital groove, the medial wall of the LHBT sheath was normal in 25%, partially torn in 39%, and completely torn in 35% (Fig. 3) (not documented in 1%). In 25 of the 218 patients (11%), a pathologic LHBT with an intact SGHL/CHL complex was observed. In these cases, the medial wall of the bicipital sheath was torn in 92% (Fig. 4). Discrepancies regarding analysis of videos by the four surgeons needed to be discussed in 11 cases to attain mutual agreement.

**Table 2:** Prevalence and type of arthroscopic lesions of the LHBT and bicipital pulley

	*N* = 218
Bicipital sheath (%)	
Normal	7.8
Partially torn	45.9
Completely torn	39.4
Not documented	6.9
SGHL/CHL complex (%)	
Normal	24.8
Stretched	38.5
Torn	35.3
Not documented	1.4
Stability of the LHBT (%)	
Centred	34.4
Subluxated	39
Dislocated	9.6
Torn	11
Not documented	6
Aspect of the LHBT (%)	
Normal	20.6
Pathologic	61.5
Not documented	6.9

**Table 3:** Relationship between aspect of the LHBT and the SGHL/CHL complex

SGHL/CHL complex LHBT	Normal (*n* = 54) (%)	Stretched (*n* = 84) (%)	Torn (*n* = 77) (%)
Normal	41	17	12
Tendinosis	46	68	66
Torn	2	9	19
Not documented	11	6	3

**Table 4:** Relationship between stability of LHBT and SGHL/CGL

SGHL/CHL complex LHBT	Normal (*n* = 54) (%)	Stretched (*n* = 84) (%)	Torn (*n* = 77) (%)
Centred	87	18	16
Subluxated	4	64	38
Dislocated	0	4	23
Completely torn	2	10	19
Not documented	7	5	4

A total of 137 LHBT tenodesis (63%) and 81 tenotomy (37%) were performed. No conservative treatment or pulley repair for the LHBT was attempted. One hundred ninety-one subscapularis were repaired according to previously described technique [7].

## Discussion

The most important finding of the present study was that in approximately 10% of cases, the bicipital reflection pulley remains intact even in the case of subscapularis tear, confirming our hypothesis. This study also showed that in cases of subscapularis tear, the SGHL/CHL complex remains intact in 25% of cases with a LHBT usually centred. Nevertheless, we observed in these patients macroscopic tendinosis or a tear of the LHBT due to a lesion of the medial wall of the bicipital sheath below the SGHL/CHL complex.

Cadaveric studies have shown the CHL to be the key ligament responsible for keeping the LHBT aligned within the tubercular groove [10, 25]. Werner et al. [32] and Habermeyer et al. [14] found that the orientation of the fibres of the SGHL towards the LHBT seemed to withstand anterior shearing forces in the lateral rotator interval, indicating that the most important function of the SGHL was the stabilization of the LHBT in its intra-articular course.

The findings of the current study have several important implications. A standard assessment of the bicipital groove with a 30° arthroscope through the posterior portal seems insufficient for fully evaluating the LHBT. Previous authors have reported that the vast majority of clinically relevant LHBT and bicipital groove lesions occur proximally and may be found and subsequently treated with standard diagnostic arthroscopic techniques [11–13, 18]. Some authors have pointed to the utility of pulling the LHBT into the joint with an arthroscopic probe through the anterior working portal. However, this manoeuvre appears insufficient based on the findings in the current study as medial sidewall lesions would be missed. Several recent researches have called into question whether arthroscopy provides an adequate view of the LHBT and bicipital groove [20, 26, 27]. Consequently, when a subscapularis tear is suspected and the SGHL/CHL complex appears normal during arthroscopic assessment, the LHBT in its intertubercular groove and the medial wall of the groove must be checked. This assessment can be performed either with an intra-articular view with the usage of a 70° arthroscope that significantly increases the length of bicipital groove visualized [24] or after opening the transverse ligament with an anterolateral extra-articular inspection (Fig. 4).

The subscapularis has a crucial role in shoulder function. First, in combination with the anterior supraspinatus, the upper subscapularis is the anterior insertion of the rotator cable [7, 29]. Second, the subscapularis stabilizes the LHBT. In an anatomical, clinical, and MRI study, Sakurai et al. [23] showed that most of the pathologic changes of the subscapularis tendon occurred on the articular side of the subscapularis tendon. In the specimens with such an articular-sided incomplete tear, the height of the medial wall of the bicipital groove was decreased, potentially being more susceptible to medial subluxation or dislocation of the LHBT. Arai et al. [1] described the “tendinous slip”. Anatomically, the most superior part of the subscapularis tendon is attached to the upper margin of the lesser tuberosity and extends as a thin tendinous slip to the fovea capitis of the humerus. The SGHL runs spirally along the biceps tendon, and histologically the SGHL attaches to the tendinous slip. The SGHL attaches to the tendinous slip of the subscapularis insertion as if it was a fold of loose connective tissue and directly supports the LHBT. The CHL, which is also a part of the same loose connective tissue, is considered to provide tension to the SGHL. The most superior insertion point of the subscapularis tendon supports the LHBT from behind the SGHL. Bennett confirmed this with an arthroscopic study that shows that the outer surface of the subscapularis tendon is intimately associated with the CHL and the SGHL with interdigitating fibres [2]. This pulley system protects the biceps tendon from subluxation. Third, it is the key to anterior elevation [4]. Finally, it has been demonstrated that subscapularis repair provides good long-term results and preservation of shoulder function [6].

According to previous studies, simple debridement, biceps tenotomy, and tenodesis concomitant to rotator cuff repair all have good clinical/functional outcomes and patient satisfaction [5, 21]. However, these studies did not focus specifically on subscapularis pathology. The results of the present study demonstrate that an action on the biceps is indicated in case of a subscapularis tear. Given that more than 90% of patients with a subscapularis tear have associated LHBT lesions or instability, we believe that preservation of the intra-articular biceps will jeopardize a subscapularis tendon repair. Indeed, Edwards et al. demonstrated that the outcomes of subscapularis repair were inferior when the biceps was preserved. Consequently, simple debridement of the biceps does not seem to be a reasonable option in the setting of subscapularis repair.

This study has several limitations. First, we did not correlate arthroscopic findings with preoperative images. Nevertheless, magnetic resonance imaging has low sensitivity, specificity, PPV, and NPV to detect this condition and arthroscopic evaluation remains the gold standard [19]. Second, the surgeries were performed by a large group of surgeons, leading to some missing values. Third, a few of the criteria used during dynamic evaluation, such as “stretch” or “subluxation”, are subjective and do not represent by definition a pathological condition and might have been interpreted differently by other surgeons. However, they are part of commonly used descriptions and have been classified in the present study under the appreciation of senior surgeons. Finally, we do not have long-term clinical outcomes or healing data to determine the best surgical option related to biceps pathology and whether subscapularis repair is systematically required. However, this study analysed prospectively the prevalence of lesions of LHBT and adjacent structures in a large cohort of patients with a subscapularis tear. The videos of all procedures were analysed by four senior surgeons, and discrepancies regarding analysis of videos were observed in only 11 cases. Although previous studies have described pathology of the LHBT, the correlations observed during this study between biceps pathology and lesions of the sheath, pulley, and SGHL/CHL complex are unique and the findings have several important implications including the necessity to address the biceps.

## Conclusion

The biceps pulley system, including the SGHL/CHL complex and subscapularis tendon, merits recognition as an important anatomical structure, and its lesions contribute to shoulder pathology. The subscapularis tendon is very important for the stability of the LHBT and should be included in the pulley system. In cases of a tear associated with a lesion of the SGHL/CHL complex, the LHBT is nearly always unstable and pathologic.

## References

[1] Arai R, Mochizuki T, Yamaguchi K, Sugaya H, Kobayashi M, Nakamura T, Akita K (2010). Functional anatomy of the superior glenohumeral and coracohumeral ligaments and the subscapularis tendon in view of stabilization of the long head of the biceps tendon. J Shoulder Elb Surg.

[2] Bennett WF (2001). Subscapularis, medial, and lateral head coracohumeral ligament insertion anatomy. Arthroscopic appearance and incidence of “hidden” rotator interval lesions. Arthroscopy.

[3] Cofield RH (1982). Subscapular muscle transposition for repair of chronic rotator cuff tears. Surg Gynecol Obstet.

[4] Collin P, Matsumura N, Lädermann A, Denard PJ, Walch G (2014). Relationship between massive chronic rotator cuff tear pattern and loss of active shoulder range of motion. J Shoulder Elb Surg.

[5] De Carli A, Vadala A, Zanzotto E, Zampar G, Vetrano M, Iorio R, Ferretti A (2012). Reparable rotator cuff tears with concomitant long-head biceps lesions: tenotomy or tenotomy/tenodesis?. Knee Surg Sports Traumatol Arthrosc.

[6] Denard PJ, Jiwani AZ, Lädermann A, Burkhart SS (2012). Long-term outcome of a consecutive series of subscapularis tendon tears repaired arthroscopically. Arthroscopy.

[7] Denard PJ, Lädermann A, Burkhart SS (2011). Arthroscopic management of subscapularis tears. Sports Med Arthrosc Rev.

[8] Dilisio M, Neyton L (2014). Comma sign—directed repair of anterosuperior rotator cuff tears. Arthrosc Tech.

[9] Ellman H (1990). Diagnosis and treatment of incomplete rotator cuff tears. Clin Orthop Relat Res.

[10] Ferrari DA (1990). Capsular ligaments of the shoulder. Anatomical and functional study of the anterior superior capsule. Am J Sports Med.

[11] Gartsman GM, Hammerman SM (2000). Arthroscopic biceps tenodesis: operative technique. Arthroscopy.

[12] Gombera MM, Kahlenberg CA, Nair R, Saltzman MD, Terry MA (2015). All-arthroscopic suprapectoral versus open subpectoral tenodesis of the long head of the biceps brachii. Am J Sports Med.

[13] Grueninger P, Nikolic N, Schneider J, Lattmann T, Platz A, Chmiel C, Meier C (2014). Arthroscopic repair of traumatic isolated subscapularis tendon lesions (Lafosse Type III or IV): a prospective magnetic resonance imaging-controlled case series with 1 year of follow-up. Arthroscopy.

[14] Habermeyer P, Magosch P, Pritsch M, Scheibel MT, Lichtenberg S (2004). Anterosuperior impingement of the shoulder as a result of pulley lesions: a prospective arthroscopic study. J Shoulder Elb Surg.

[15] Hartzler RU, Burkhart SS (2014). Medial biceps sling takedown may be necessary to expose an occult subscapularis tendon tear. Arthrosc Tech.

[16] Jost B, Koch PP, Gerber C (2000). Anatomy and functional aspects of the rotator interval. J Shoulder Elb Surg.

[17] Lo IK, Burkhart SS (2003). The comma sign: an arthroscopic guide to the torn subscapularis tendon. Arthroscopy.

[18] Mall NA, Chahal J, Heard WM, Bach BR, Bush-Joseph CA, Romeo AA, Verma NN (2012). Outcomes of arthroscopic and open surgical repair of isolated subscapularis tendon tears. Arthroscopy.

[19] Momenzadeh OR, Gerami MH, Sefidbakht S, Dehghani S (2015). Assessment of correlation between MRI and arthroscopic pathologic findings in the shoulder joint. Arch Bone Jt Surg.

[20] Moon SC, Cho NS, Rhee YG (2015). Analysis of “hidden lesions” of the extra-articular biceps after subpectoral biceps tenodesis: the subpectoral portion as the optimal tenodesis site. Am J Sports Med.

[21] Oh JH, Lee YH, Kim SH, Park JS, Seo HJ, Kim W, Park HB (2016). Comparison of treatments for superior labrum-biceps complex lesions with concomitant rotator cuff repair: a prospective, randomized, comparative analysis of debridement, biceps tenotomy, and biceps tenodesis. J Arthrosc Relat Surg.

[22] Patte D (1990). Classification of rotator cuff lesions. Clin Orthop Relat Res.

[23] Sakurai G, Ozaki J, Tomita Y, Kondo T, Tamai S (1998). Incomplete tears of the subscapularis tendon associated with tears of the supraspinatus tendon: cadaveric and clinical studies. J Shoulder Elb Surg.

[24] Sheean AJ, Hartzler RU, Denard PJ, Lädermann A, Hanypsiak BT, Burkhart SS (2016). A 70 degrees arthroscope significantly improves visualization of the bicipital groove in the lateral decubitus position. Arthroscopy.

[25] Slatis P, Aalto K (1979). Medial dislocation of the tendon of the long head of the biceps brachii. Acta Orthop Scand.

[26] Taylor SA, Fabricant PD, Bansal M, Khair MM, McLawhorn A, DiCarlo EF, Shorey M, O’Brien SJ (2015). The anatomy and histology of the bicipital tunnel of the shoulder. J Shoulder Elb Surg.

[27] Taylor SA, Khair MM, Gulotta LV, Pearle AD, Baret NJ, Newman AM, Dy CJ, O’Brien SJ (2015). Diagnostic glenohumeral arthroscopy fails to fully evaluate the biceps-labral complex. Arthroscopy.

[28] Toussaint B, Barth J, Charousset C, Godeneche A, Joudet T, Lefebvre Y, Nove-Josserand L, Petroff E, Solignac N, Hardy P, Scymanski C, Maynou C, Thelu CE, Boileau P, Graveleau N, Audebert S, French Arthroscopy S (2012). New endoscopic classification for subscapularis lesions. Orthop Traumatol Surg Res.

[29] Visona E, Cerciello S, Godeneche A, Neyton L, Fessy MH, Nove-Josserand L (2015). The “comma sign”: an anatomical investigation (dissection of the rotator interval in 14 cadaveric shoulders). Surg Radiol Anat.

[30] Walch G, Nove-Josserand L, Boileau P, Levigne C (1998). Subluxations and dislocations of the tendon of the long head of the biceps. J Shoulder Elb Surg.

[31] Walch G, Nove-Josserand L, Levigne C, Renaud E (1994). Tears of the supraspinatus tendon associated with “hidden” lesions of the rotator interval. J Shoulder Elb Surg.

[32] Werner A, Mueller T, Boehm D, Gohlke F (2000). The stabilizing sling for the long head of the biceps tendon in the rotator cuff interval. A histoanatomic study. Am J Sports Med.

